# Complete Genome Sequence of *Achromobacter* Strain ES-001, a Betaproteobacterium Associated with a Cellulolytic Soil Community

**DOI:** 10.1128/MRA.00828-21

**Published:** 2021-10-07

**Authors:** Chamil E. Fernando, Joseph J. LaForge, Stephen D. Fields

**Affiliations:** a Department of Biological Sciences, Emporia State University, Emporia, Kansas, USA; Montana State University

## Abstract

The genome sequence of the soilborne bacterium *Achromobacter* strain ES-001, assembled from Illumina NextSeq and Nanopore MinION reads, is rich in genes predicted to encode iron, arsenic, and hydrocarbon metabolism, as well as type 6 secretion components. The sequenced genome will aid in determining the roles of noncellulolytic species in cellulose-enriched environments.

## ANNOUNCEMENT

The Gram-negative genus *Achromobacter* is composed of motile, facultative aerobes that generally inhabit soils and water (1), with some species also acting as opportunistic pathogens (2–4). *Achromobacter* strain ES-001 is associated with microbial communities of cellulose-rich soils and can be maintained on carboxymethylcellulose (CMC) (5). Cellulolytic activity, however, has not been previously observed in the genus. To better understand its metabolic contributions to cellulolytic soil communities, the complete genome sequence of *Achromobacter* strain ES-001 is presented here.

Soil-water suspensions (1:10) from leaf-mulched soil in east central Kansas, USA (38.43 N, 96.21 W) were plated onto M9 agar (Sigma-Aldrich, St. Louis, MO, USA) with 1% CMC (Sigma-Aldrich) as the carbon source (5). After 10 passages to fresh M9/CMC agar at 30°C, single colonies of an unknown species, designated ES-001, were isolated from the streak plates to M9/CMC plates and maintained thereafter on LB agar (6) at 30°C. DNA from cultures grown at 30°C at 250 rpm in LB broth was isolated using the Quick-DNA fungal/bacterial miniprep kit (Zymo Research, Irvine, CA, USA). The University of Kansas Genome Sequencing Core Laboratory (Lawrence, KS, USA) prepared a genomic library using the NEBNext Ultra II DNA library prep kit (New England Biolabs, Ipswich, MA, USA), targeting 200-bp fragments. Illumina NextSeq 550 sequencing with a 300-cycle midoutput kit v2.5 yielded 23,450,192 paired-end reads, for a total of 3.542 Gb of sequence with a mean quality score of Q34.54. The Oxford Nanopore ligation sequencing kit (SQK-LSK109, Oxford Nanopore, Oxford, UK) was used to prepare a second library from the same DNA as above, which was sequenced with the Nanopore MinION platform to generate 40,000 reads with an average length of 6,638 bp. The Illumina and Nanopore reads were uploaded to the public usegalaxy.org server (7) for quality analysis and processing using fastp v0.19.5+galaxy1 and Porechop v0.2.3 tools (8, 9) with default settings. Unicycler v0.4.8.0 (10) was used to assemble 3.38 Gb of paired Illumina reads (516.8-fold coverage), with 25,303 high-quality MinION reads (170.38 Mb, 26.0-fold coverage) as scaffolds, to generate a single, circular 6,544,928-bp contig with 64.5% GC content. Nanopore reads spanning the ends of the assembled contig confirmed circularity.

The assembled ES-001 16S rRNA gene, when used as the query in BLASTN searches of the NCBI 16S rRNA database and in Needleman-Wunsch alignments, is 100% identical to Achromobacter spanius NTCT13519 (GenBank accession number LR134302.1). However, assessment of the average nucleotide identity of orthologs using the Rapid Annotation Subsystems Technology (RAST) v2.0 SEED (11) and OrthoANIu (12) servers showed that ES-001 is 87.9% identical to *A. spanius* NTCT13519, well below the proposed species cutoff of 96% (13). Therefore, *Achromobacter* strain ES-001 may be a distinct species.

NCBI PGAP (Prokaryotic Genome Annotation Pipeline) annotation (14) predicted 5,766 protein-coding genes, 74 RNA-coding genes, and 95 pseudogenes. Predicted genes for auxin synthesis, iron acquisition, hydrocarbon metabolism, arsenic/copper/heavy metal regulation, and a complete type 6 secretion system (T6SS) (15) suggest that *Achromobacter* strain ES-001 performs roles in stimulating plant growth, protecting against environmental toxins, and regulating microbial community composition, similar to the environmental roles of other *Achromobacter* species (16–20).

### Data availability.

The *Achromobacter* strain ES-001 genome sequence has been deposited in DDBJ/ENA/GenBank under accession number CP079940. Raw sequence data used for assembly are deposited in DDBJ/ENA/GenBank under SRA accession number SAMN20284516. The assembly described in this paper is the first version, CP079940.1.
